# A Retrospective Observational Study Evaluating the Synergistic Effect of a Novel Combination of Alfapin + Native Type 2 Collagen + Mobilee (Hyaluronic Acid) + CurQlife (Curcumin) Nutraceuticals in the Symptomatic Improvement of Knee Osteoarthritis

**DOI:** 10.7759/cureus.36123

**Published:** 2023-03-14

**Authors:** Yogeesh D Kamat, Bishwaranjan Das, Kandarp Thakkar, Manish Mahajan

**Affiliations:** 1 Orthopedics, Hip & Knee Joint Replacement, Sports Injury, Kasturba Medical College Hospital, Mangalore, IND; 2 Physiotherapy (Hip, Knee & Sports injury) at Comprehensive Knee & Hip Care Centre, Kasturba Medical College Hospital, Mangalore, IND; 3 Medical Affairs, Zydus Lifesciences, Ahmedabad, IND

**Keywords:** clagen, nutraceuticals, vas score, koos, osteoarthritis

## Abstract

Background

Treatment of pain and inflammation form the mainstay of osteoarthritis (OA) management. Non-steroidal anti-inflammatory drugs (NSAIDs), due to their inflammation-blocking mechanism, are a highly effective class of drugs for chronic pain and inflammation in OA. However, this comes at a cost of increased risk for multiple adverse effects, including gastrointestinal bleeding, cardiovascular side effects, and NSAID-induced nephrotoxicity. To minimize the potential risk of an adverse event, numerous regulatory bodies and medical societies recommend using the lowest effective NSAID dose for the shortest time necessary. One potential strategy to achieve this is the use of disease-modifying osteoarthritis drugs (DMOADs) containing anti-inflammatory and analgesic properties instead of NSAIDs for the management of OA. This study focuses on the efficacy of Clagen™ [Aflapin (*Boswellia serrata* extract) + native type 2 collagen + Mobilee (hyaluronic acid (60-70%), polysaccharides (>10%), and collagen (>5%)) + CurQlife (Curcumin)] for the symptomatic improvement in OA patients as well as if this combination is effective in the long-term management of OA instead of NSAIDs.

Methodology

In this retrospective observational study, a total of 300 patients were screened, of whom 100 OA patients who fulfilled the criteria and agreed to be part of the study were enrolled. The data were analyzed to evaluate the efficacy of the nutraceutical formulation Clagen™ in patients with OA of the knee. From the baseline to two months, primary outcomes of improvement in the Visual Analog Scale (VAS) score, range of motion, and Knee Injury and Osteoarthritis Outcome Score (KOOS) were measured at monthly follow-up. Statistical analyses were performed according to the results obtained from the parameters. The tests were performed at a 5% significance level (p <0.05). The qualitative characteristics were described using absolute and relative frequencies, and the quantitative measures were described as summary measures (mean, standard deviation).

Results

Of the 100 patients enrolled in the study, 99 (64 males and 35 females) completed the study. The mean age of the patients was 50.6 ± 13.9 years, and the mean body mass index was 24.5 ± 3.5 kg/m^2^. The statistical analysis of the outcomes from the baseline to the two-month follow-up was analyzed using paired t-test. The difference in the mean of VAS pain score at baseline and two months was 3.3 ± 1.8 [t (97) = 18.2; p < 0.05], which showed a significant reduction in pain at two months. Moreover, the difference in the mean of the goniometer value of 7.3 ± 7.3 [t (98) = -10.0, p < 0.05] indicated statistically significant improvements in the range of motion. It was also observed that Clagen™ significantly improved the composite KOOS score by 10.8% at the end of two months. Similarly, KOOS scores for Symptoms, Function, and Quality of Life showed improvements of 9.6%, 9.8%, and 7.8%, respectively, and were statistically significant (p < 0.05).

Conclusions

Clagen™ exerted positive adjuvant effects in the management of OA. The combination not only improved the symptoms and quality of life but, in the light of future perspective, NSAIDs can be withdrawn in OA patients, considering their long-term negative effects. To validate these findings further long-term studies with a comparison arm of NSAIDs are needed.

## Introduction

Osteoarthritis (OA) is a progressive degenerative disease characterized by inflammation of cartilage and synovium that causes joint stiffness, swelling, pain, and loss of mobility [1]. The overall prevalence of knee OA was 28.7% in India according to a 2016 study. Women are at high risk for OA, with a prevalence rate of 31.6%. However, obesity, advanced age, and sedentary work also contribute to OA [2]. Because OA is a complex and multifactorial disease, with an evolution in pharmaceutical science, the life expectancy of individuals with OA has increased. Therefore, OA is considered one of the most significant causes of disability in adults above 50 years of age [1]. Kellgren-Lawrence classification is widely used for OA grading, ranging from grade 0 (no presence of OA), grade 1 (doubtful), grade 2 (mild, loss of cartilage), grade 3 (moderate, joint space reduction), to grade 4 (severe) [3]. Among various therapeutic approaches to OA, the first line of therapy is symptomatic treatment, where non-steroidal anti-inflammatory drugs (NSAIDs) and opioid analgesics are used to relieve pain [4]. However, NSAIDs may cause gastrointestinal ulcers, hypertension, serious cardiovascular events, acute renal failure, and worsening of preexisting heart failure, restricting the long-term use of NSAIDs [5]. Some studies discourage the prescription of opioid analgesics because of their risk and serious adverse effects. However, some studies have also concluded that treatment with opioids is not superior to treatment with non-opioid analgesic medications over 12 months [6]. Cases that get worse over time may require surgery. However, other approaches include lifestyle modification and the use of nutraceuticals [7].

Nutraceuticals are dietary compounds that play an important role in the catabolic and anabolic processes that occur in chondrocytes and synovial fluid within the articular cartilage. In OA, the structural integrity of articular cartilage is impaired [1]. Due to their good efficacy and safety, there is an emphasis on using nutraceuticals. The efficacy of nutraceuticals in reducing OA pain is evaluated based on the Visual Analog Scale (VAS) score, range of motion, and Knee Injury and Osteoarthritis Outcome Score (KOOS). Among the myriad nutraceuticals used in OA, chondroitin sulfate, glucosamine sulfate, collagen, hyaluronic acid, Aflapin, curcumin, and methylsulfonylmethane have shown impressive results in the improvement of clinical symptoms, reducing pain and decreasing inflammation associated with OA [7-9].

The presence of anti-type II collagen antibodies in osteoarthritic cartilage further supports the idea that immunological mechanisms play a role in the progression of OA. When undenatured collagen type II is orally administered, its epitopes interact with gut-associated lymphoid tissue (GALT) in the duodenum. This interaction induces oral tolerance to antigens and reduces the attack of T cells on cartilage throughout the body by presenting active epitopes with the correct three-dimensional structures as GALT. Small doses of glycosylated undenatured collagen type II help to develop immune tolerance. This process also helps prevent attacks by killer T cells [10].

Undenatured collagen type II helps increase the functionality and mobility of the joints and alleviates pain in OA patients [4]. Mobilee is rich in hyaluronic acid, which is a component of synovial fluid, enhances chondrocyte synthesis, prevents cartilage degradation, and promotes its regeneration [11]. Aflapin supplementation reduces the circulating matrix metalloproteinase 3 (MMP-3), tumor necrosis factor-alpha, high-sensitivity C-reactive protein, and marker of type II collagen cleavage, which affirms that Aflapin is clinically efficacious, fast-acting, and safe in treating OA with no significant adverse effects observed [8]. Curcumin downregulates the catabolic and degradative effects in chondrocytes and inhibits the production of MMP-3, MMP-9, and MMP-13, which shows the anti-arthritic potential of curcumin [9].

According to previously published data, all components show good efficacy and safety in the treatment of OA when administered solely or in a combination of the two. This study aims to evaluate the synergistic effects of all four components of Clagen™ [Aflapin (*Boswellia serrata* extract) (0.01 g) + native type 2 collagen (0.04 g) + Mobilee [hyaluronic acid (60-70%), polysaccharides (>10%), and collagen (>5%)] (0.04 g) + CurQlife (Curcumin) (0.01 g)] for the symptomatic improvement of OA patients, as well as to understand if this combination is effective in the long-term management of OA as an alternative to NSAIDs. The dose of Clagen™ is one capsule a day for three to six months in OA patients.

## Materials and methods

Participants

Patients of either gender, between 21 and 75 years of age, and diagnosed with knee OA were included in the study. Patients with unilateral or bilateral knee OA for more than two months confirmed by a radiologist, i.e., X-rays showing small osteophyte formation (because this is a real-world analysis data so this study is single arm bias), those with joint space reduction, those who could walk, those able to give informed consent, and those available for the duration of the study period (two months) were included in the study. Patients using other therapies to alleviate pain in OA, such as exercise, heat/cold therapy, joint protection, and physiotherapy/occupational therapy, and patients whose Kellgren-Lawrence radiological stage was I, II, or III were included in the study.

Patients with a history of underlying inflammatory diseases such as septic arthritis, gout, pseudogout, rheumatoid arthritis, inflammatory joint disease, Paget’s disease, joint fracture, fibromyalgia, Wilson’s disease, acromegaly, ochronosis, heritable arthritic disorder or collagen gene mutations, hyperuricemia (uric acid >7.2 mg/dL), expected surgery in the next few months, recent injury in the area affected by OA of the knee, a history of cancer, a history of psychiatric disorder that may impair the ability to provide written informed consent, use other natural health products including *Boswellia *and Aflapin one month before and during the study, use of other multivitamins and mineral supplements containing vitamins and minerals as the sole ingredients of Clagen™, comorbidities that adversely affect patient’s ability to complete the study or its measures were excluded from the study.

Study design and treatments

**Figure 1:**
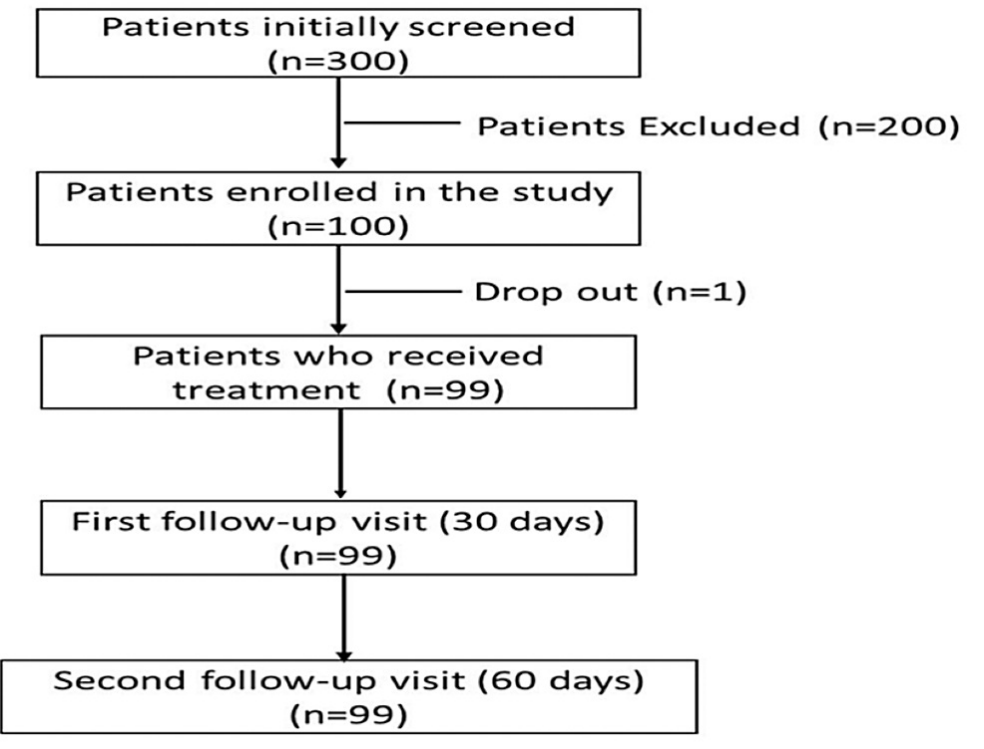
Patient disposition.

This retrospective observational study screened 300 patients for six months of either gender and collected data from 100 patients based on the inclusion and exclusion criteria to evaluate the efficacy of the nutraceutical formulation, Clagen™, in patients with OA of the knee. The ethical approval was waived by the local ethics committee because it was a retrospective study, and the follow-up was done at the end of month two. The data captured were evaluated for pre-treatment objective evaluation on day zero and post-treatment objective evaluation at one month and two months (Figure 1).

Assessment

Functional disability was assessed at baseline and all follow-up visits (days 0, 30, 60) by the investigators. Pain, stiffness, and physical function were assessed using a goniometer, KOOS, and VAS scores. The pain, stiffness, and function subscales of the KOOS and VAS were normalized to a scale of 0-100 units. The knee joint range of motion of the test leg was quantified using an 18-inch flexible and adjustable plastic goniometer commonly employed by doctors in hospitals. Analyses of these endpoints were based on the time-weighted average change from baseline over 30 to 60 days.

Primary outcome

To assess the efficacy of Clagen™ outcome measurements, the primary endpoint was a decrease in the VAS score and an increase in KOOS and goniometer values (range of motion) from baseline to month two.

Statistical analysis

Outcome variables were assessed for conformance to the normal distribution (done with the help of histograms) and transformed as required. For statistical analysis, a t-test (SAS university edition; SAS Institute Inc., Cary, NC, USA) was used with p-values <0.05 to compare the means at month two. It is one of the most widely used statistical hypothesis tests in pain studies.

## Results

The baseline characteristics of OA patients enrolled in the study are presented in Table 1.

**Table 1:** Baseline characteristics of OA patients enrolled in the study. BMI: body mass index; VAS: Visual Analog Scale; OA: osteoarthritis; KOOS: Knee Injury and Osteoarthritis Outcome Score

Demographics	N = 99
Gender (male) (n, %)	64 (64.5%)
Age (mean ± SD, range)	50.6 ± 13, 21–74
Weight (mean ± SD, range)	65.1 ± 10.7, 43–92
Height (mean ± SD, range)	162.9 ± 8.5
BMI (mean ± SD)	24.5 ± 3.5
Grading of OA	
OA grade 1 (n, %)	37 (37.3%)
OA grade 2 (n, %)	35 (35.3%)
OA grade 3 (n, %)	18 (18.1%)
OA grade 4 (n, %)	8 (8.0%)
Goniometer (mean ± SD)	112.3 ± 11.8
VAS (mean ± SD)	6.5 ± 1.6
KOOS (mean ± SD)	54.9 ± 20.5

The mean values of outcomes from baseline to the two-month follow-up are presented in Table 2.

**Table 2:** Mean value of participants’ VAS, KOOS, and goniometer score reading at baseline and month two. VAS: Visual Analog Scale; KOOS: Knee Injury and Osteoarthritis Outcome Score

	Scale	Baseline	At the end of month two	Percentage improvement
Goniometer	Range of motion knee flexion (in degree)	112.3 ± 11.8	119.6 ± 10.2	6.5% (p < 0.05)
	Range of motion knee extension (in degree)	6.5 ± 3.4	3 ± 4.7	53.8% (p < 0.05)
VAS (Pain)		6.5 ± 1.6	3.3 ± 2.5	49.3% (p < 0.05)
KOOS		54.9 ± 21.1	63.5 ± 24.5	15.7% (p < 0.05)

**Figure 2:**
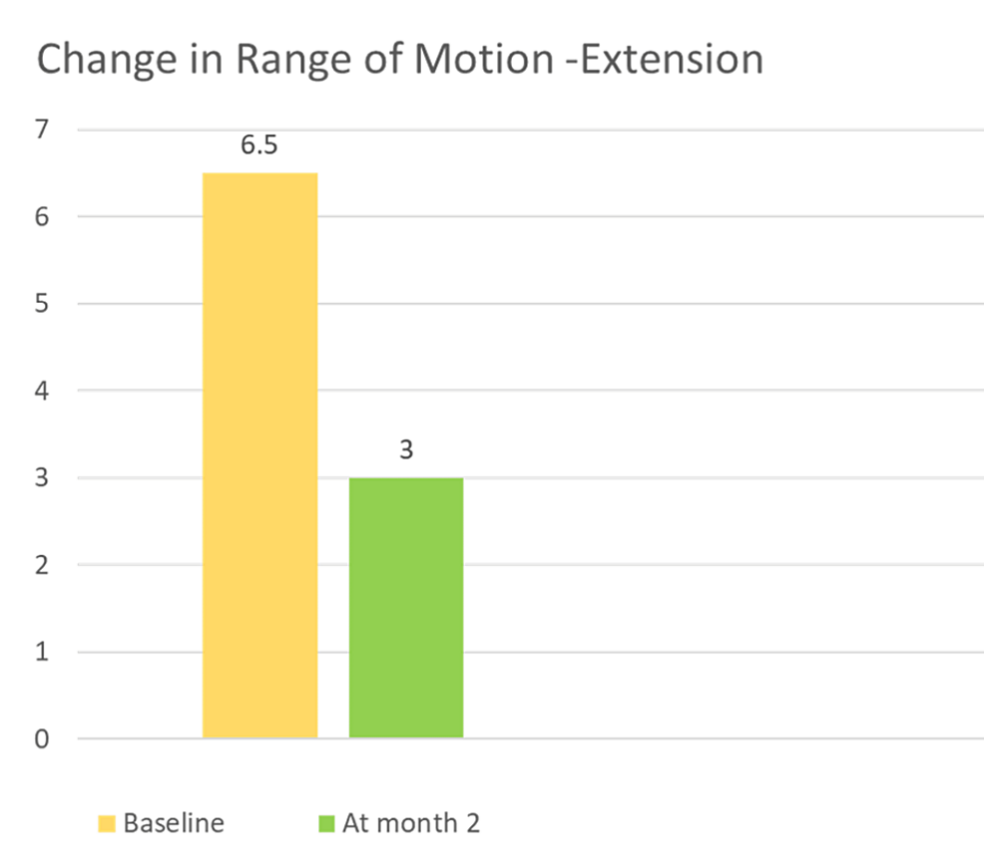
Change in the range of motion (extension) at baseline and month two.

Improvement was observed in the range of motion during extension from baseline and at the two-month follow-up (Figure 2).

**Figure 3:**
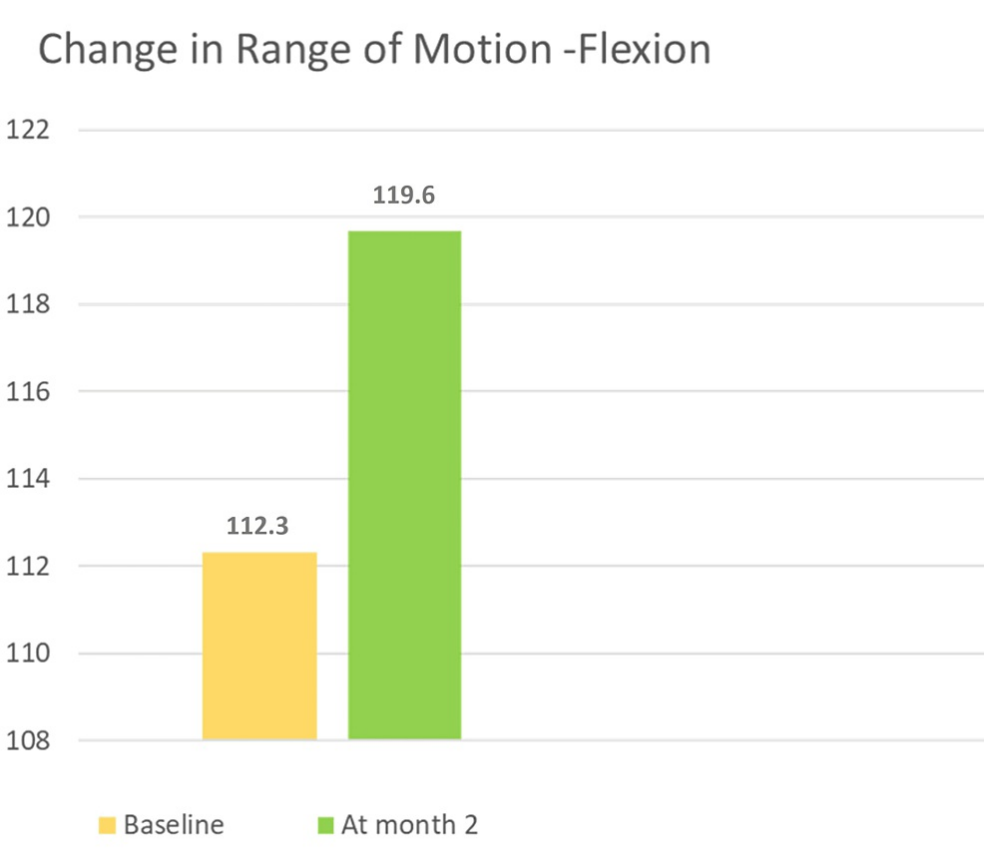
Change in the range of motion (flexion) at baseline and month two.

Improvement was observed in the range of motion during flexion from baseline and at the two-month follow-up (Figure 3).

**Figure 4:**
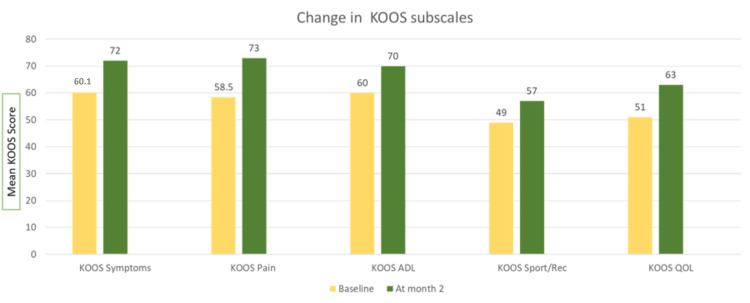
Change in KOOS value at baseline and month two. ADL: Activities of daily living; QOL: quality of life; KOOS: Knee Injury and Osteoarthritis Outcome Score

Improvement was observed in the KOOS subscales such as KOOS symptoms (p < 0.05), KOOS pain (p < 0.05), KOOS quality of life (p < 0.05), KOOS activities of daily living (p < 0.05), and KOOS sports (p < 0.05) from baseline and at the two-month follow-up (Figure 4).

**Figure 5:**
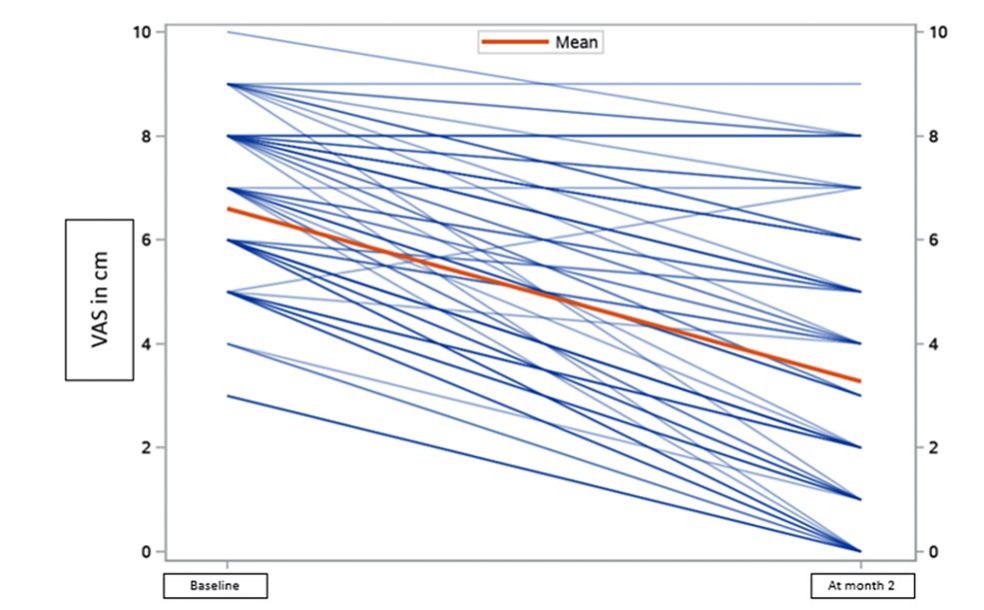
VAS score. VAS score of patients on Clagen™ at baseline and month two. VAS: Visual Analog Scale

**Figure 6:**
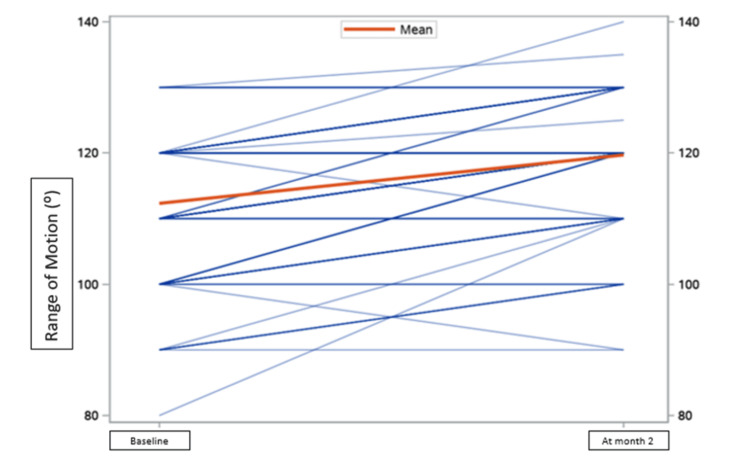
Range of motion. Range of motion in patients on Clagen™ at baseline and month two.

**Figure 7:**
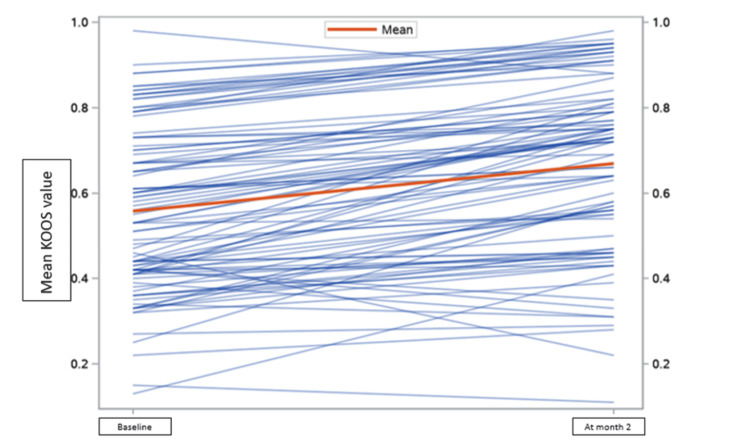
KOOS score. KOOS score of patients on Clagen™ at baseline and month two. KOOS: Knee Injury and Osteoarthritis Outcome Score

The difference in the mean of the VAS score and range of motion to measure primary outcomes at baseline and two months was 3.3 ± 1.8 [t (97) = 18.2; p < 0.05] and 7.4 ± 7.3 [t (98) = -10.0; p < 0.05], respectively, and KOOS score was significantly improved by 10.8% at the end of two months, which showed a significant reduction in pain compared to baseline and at the two-month follow-up (Figures 5-7).

## Discussion

The primary objective of this retrospective observational study was to evaluate the efficacy of Clagen™ on knee OA. Adding all these four components to the standard product resulted in a clear benefit in terms of clinical improvements, such as KOOS, VAS scores, and goniometer values.

Although the exact etiology of OA is unknown, it is associated with inflammation in articular cartilage, which can cause abnormal joint structure in the knee and hip and is accompanied by pain. The most prescribed treatments are analgesics, NSAIDs, and glucosamine and chondroitin sulfate but these drugs have serious adverse effects on the gastrointestinal tract and cardiovascular system. Moreover, glucosamine and chondroitin sulfate cannot be prescribed to patients suffering from asthma, prostate cancer, hyperlipidemia, or hemophilia. The combination recommended by Osteoarthritis Research Society International (OARSI) has myriad side effects; however, preparations such as nutraceuticals do not have serious side effects. Therefore, nutraceutical treatments that may mitigate pain and inflammation and can be prescribed to every patient have been investigated as potential primary or adjunct therapies for relieving arthritis symptoms. Low-dose, short-term NSAIDs and pharmaceutical-grade glucosamine and chondroitin sulfate are recommended by the European Society for Clinical and Economic Aspects of Osteoporosis, Osteoarthritis and Musculoskeletal Diseases (ESCEO) whereas OARSI strongly recommends against their use [12]. Our result was similar to the effects of other studies published so far.

This study assesses the combined effects of all four components of Clagen™ because each component showed good efficacy and safety when prescribed solely. Some systematic reviews and meta-analyses have suggested that 8-12 weeks of standardized turmeric extract (curcumin) treatment may reduce arthritis symptoms and result in similar improvements of the symptoms as NSAIDs such as ibuprofen and diclofenac sodium. Therefore, curcumin can be cautiously recommended for alleviating the symptoms of arthritis, especially OA [13]. *Boswellia* extract (Aflapin) showed anti-inflammatory effects on chondrocytes during in vitro and ex vivo experiments. Boswellic acid significantly reduces the infiltration of leukocytes in the knee joint, reducing inflammation and preventing the degradation of type II collagen, which improves the Western Ontario and McMaster Universities Osteoarthritis Index (WOMAC) score, which is a scale to assess pain intensity. The results of several randomized, placebo-controlled studies also suggest that *B. serrata* extracts (Aflapin) are effective and safe alternative interventions for the treatment of OA [14]. In our study, both compounds were used, and they showed a positive result in terms of VAS and the goniometer scale.

It has been found that native type II collagen is effective in reducing arthritic pain in animal models and improving symptoms in patients with rheumatoid arthritis. However, few studies have evaluated the efficacy of native type II collagen in treating OA. Type II collagen (40 mg/day) reduced the WOMAC and improved VAS scores more effectively [15]. A pilot study demonstrated that methylsulfonylmethane and Mobilee have a beneficial effect on OA chondrocytes metabolism, probably due to the modulation of the nuclear factor kappa B pathway, advocating the use of these substances in OA treatment [16].

A study assessing the effects of an oral preparation containing hyaluronic acid on osteoarthritic knee joint pain found that the combination of hyaluronic acid and other glycosaminoglycans is safe and effective for the treatment of patients with knee OA [17]. These results are similar to our study regarding patients’ quality of life.

All components of Clagen™ reduce the symptoms of OA and improve the patient’s quality of life. To our knowledge, this is the first study evaluating the synergistic effects of Aflapin, curcumin, native type II collagen, and hyaluronic acid. The present study demonstrates the potential of Clagen™ in alleviating pain and joint stiffness and improving physical function in OA patients. Pain, stiffness of joints, and physical discomfort are the major clinical manifestations of OA. Side effects of components of Clagen™ such as oral hyaluronic acid include nausea, vomiting, and gastrointestinal discomfort; Aflapin results in headache; and curcumin causes diarrhea, headache, rash, and yellow stool. However, no side effects were reported while assessing the combined effect of all four components of Clagen™ in this study [18-20].

Limitations of the present study are a small sample size, a short follow-up duration, and the lack of a comparison arm.

## Conclusions

Clagen™ exerted positive adjuvant effects in the management of OA. The combination not only improved the symptoms and quality of life but, in the light of future perspectives, NSAIDs can be withdrawn in OA patients, considering their long-term negative effects. To validate these findings further long-term studies with a comparison arm of NSAIDs are needed.
